# Supplementary figures and images for: A Novel Antithrombocytopenia Agent, Rhizoma cibotii, Promotes Megakaryopoiesis and Thrombopoiesis through the PI3K/AKT, MEK/ERK, and JAK2/STAT3 Signaling Pathways
Source: Int J Mol Sci. 2022 Nov 14;23(22):14060. doi: 10.3390/ijms232214060 (PMC9694118; doi:10.3390/ijms232214060)

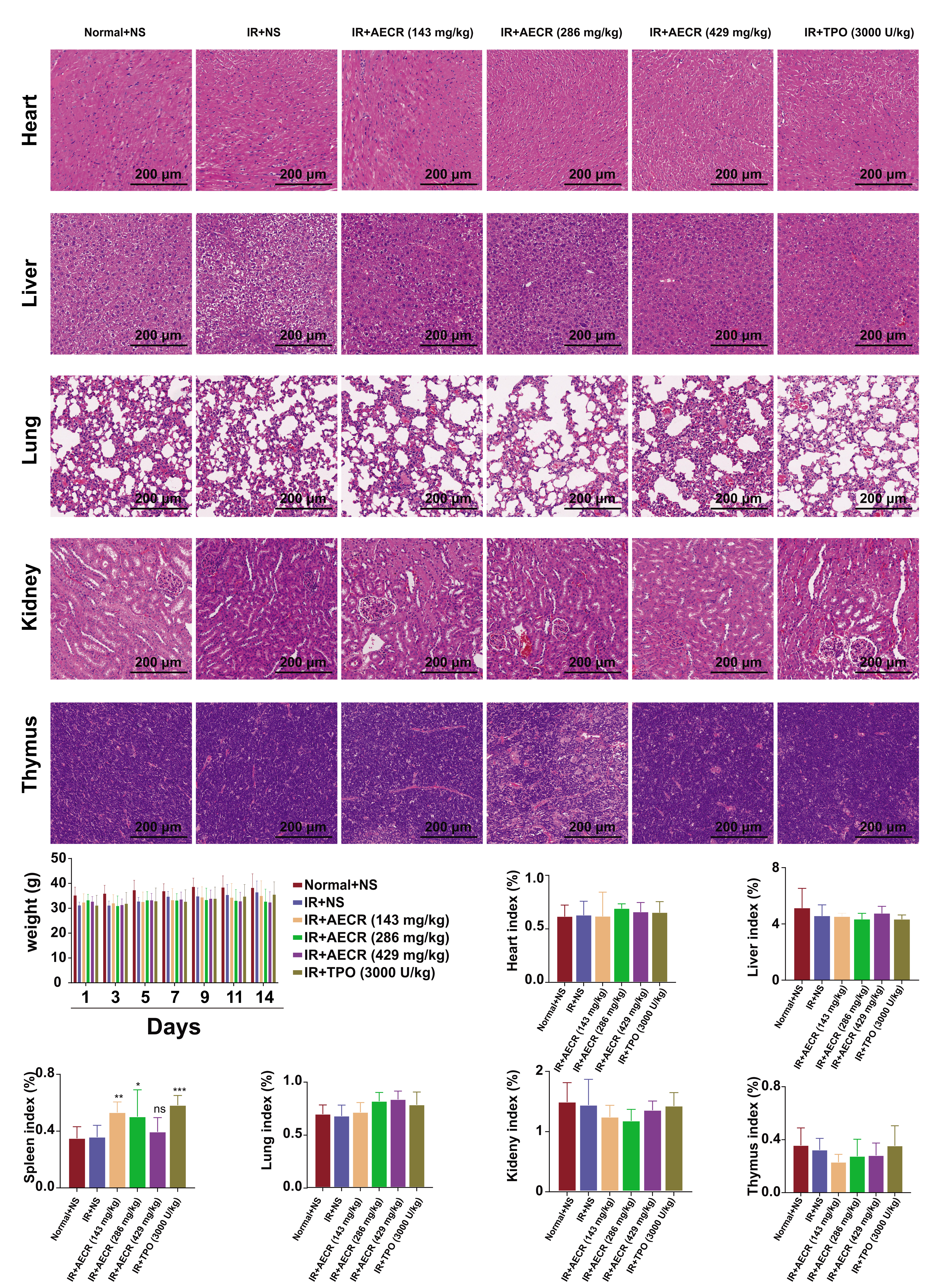

Supplement: Supplementary file 1 [file ijms-23-14060-s001.zip › Figure S1.jpg]
